# Assessment of Chilling Injury in Boar Spermatozoa by Kinematic Patterns and Competitive Sperm-Oviduct Binding In Vitro

**DOI:** 10.3390/ani12060712

**Published:** 2022-03-11

**Authors:** Heiko Henning, Jennifer Franz, Julia Batz-Schott, Xuyen Le Thi, Dagmar Waberski

**Affiliations:** 1 Institute of Farm Animal Genetics, Friedrich-Loeffler-Institut, Höltystraße 10, 31535 Neustadt am Rübenberge, Germany; heiko.henning@fli.de; 2 Unit for Reproductive Medicine, Clinic for Pigs and Small Ruminants, University of Veterinary Medicine Hannover, Bünteweg 15, 30559 Hannover, Germany; jenfranz@gmx.de (J.F.); batzjulia@googlemail.com (J.B.-S.); xuyen.lethi@web.de (X.L.T.)

**Keywords:** boar semen, semen preservation, motility, sperm reservoir, oviduct explant, chilled semen, cluster analysis

## Abstract

**Simple Summary:**

Artificial insemination is widely used for pig reproduction. Boar spermatozoa for use in artificial insemination are usually preserved at 17 °C for several days. Storage at lower temperature would be beneficial to reduce the risk of bacterial growth but is hindered by the high chilling sensitivity of boar spermatozoa. Currently, new preservation concepts are evolving which aim to store boar sperm at 5 °C. Before introducing these for animal breeding, sensitive tests are necessary to detect chilling-induced sperm damage. The aim of the study was to examine the potential of two tests systems for detecting sperm injury after chilling in two different semen extenders. It was shown that the analysis of sperm movement patterns and the binding of sperm to oviductal tissue sensitively indicate subtle differences of semen extenders to preserve the quality of chilled spermatozoa. The application of these tests is recommended for testing new hypothermic preservation strategies for boar semen.

**Abstract:**

Sensitive detection of chilling injury in boar spermatozoa is required to evaluate novel hypothermic preservation concepts. The study’s aim was to examine whether analyses of motility patterns and sperm binding in a competitive oviduct explant assay (cOEA) sensitively detect chilling-induced alterations in sperm function. Semen samples (*n* = seven boars) were split into four subsamples by dilution either in Beltsville Thawing Solution (BTS) or Androstar^®^ Plus and stored at 5 °C or 17 °C. Storage temperature had a significant effect on the distribution of spermatozoa in seven major kinematic clusters. The effect size of chilling at 5 °C as estimated by Cramer’s V was higher (*p* < 0.05) in the BTS medium (0.21) compared to AndroStar^®^ Plus (0.11). Spermatozoa extended in Androstar^®^ Plus had higher relative binding capacity compared to sperm in BTS (*p* < 0.05). Binding indices correlated with the percentage of viable, acrosome-intact (r = 0.62) and motile spermatozoa (r = 0.72, both *p* < 0.001). The cluster size of sperm with slow, vigorous movement was negatively correlated with sperm-oviduct binding (r = −0.43, *p* < 0.05). In conclusion, the cluster analysis of sperm kinematics and competitive sperm oviduct binding *in vitro* present meaningful biological tests to assess novel concepts for hypothermic semen preservation.

## 1. Introduction

Artificial insemination (AI) is a well-established reproductive biotechnology in pigs worldwide. In contrast to other livestock, spermatozoa of boars are highly sensitive to chilling injury and therefore are commonly stored between 15 °C and 18 °C. The relatively high storage temperature bears a risk for bacterial growth which is controlled by adding antibiotics to semen extenders. Currently, new concepts for hypothermic preservation of boar semen at 5 °C are evolving to reduce the usage of antibiotics in AI in pigs [1,2]. Assessing chilling injury requires sensitive *in vitro* assays which test for traits that identify physiological meaningful changes in sperm function.

Chilling, like other effectors of semen handling, induces cell death in a certain subpopulation of fragile sperm and affects cell function in other cohorts on a sublethal level [3,4,5]. A basic sperm function is motility, this being essential to overcome the barriers of the female reproductive tract and to reach the oocytes. Given the heterogenous nature of sperm in a semen sample, it is likely that spermatozoa respond differently to the chilling stress. Spermatozoa probably require *in vivo* an active, vigorous flagellar beating that generates sufficient force to passage the mucus and narrow folds of the utero-tubal junction, and that later contributes to the sperm’s ascent to the fertilization site [6,7]. Although it remains unclear which motility pattern(s) are exactly required for these processes *in vivo*, computer-assisted semen analysis (CASA) of sperm kinematics in diluted boar semen is able to identify motility descriptors that are related to fertility measures [8,9]. A considerable heterogeneity in the movement patterns of individual spermatozoa is intrinsic to semen samples. Characterizing such sperm heterogeneity by cluster analysis procedures of kinematic parameters derived from computer-assisted sperm motility analysis is a tool that provides valuable information on male reproductive performance [10,11]. Notably, the subpopulation structure of semen samples can be modified by semen handling and/or storage (e.g., [12,13,14,15]). Identifying changes in the movement pattern of motile sperm in comparison to samples from well-established protocols therefore will help to critically evaluate the effects of hypothermic semen preservation strategies or accidental chilling during transport of semen portions.

Upon reaching the lower oviductal isthmus, sperm binding to the epithelial lining to establish the female sperm reservoir is yet another critical step for fertilization success, particularly if (artificial) insemination takes place several hours or days before ovulation. The binding of the spermatozoa to the oviduct epithelial cells requires viable, non-capacitated spermatozoa with functionally intact binding sites for glycans on their plasma membrane [16,17,18]. It has been shown that testing boar sperm binding to oviduct explants or epithelial cell aggregates *in vitro* is indicative of the relative fertility level of individual males [19,20,21]. Oviductal explants are mostly collected from tissues of several slaughtered sows with unknown fertility history. To reduce the bias of the individual explant/aggregate on the *in vitro* assays, we recently established a competitive oviduct explant assay (cOEA). The cOEA allows the direct comparison of semen treatment effects under identical conditions by using differentially fluorescent tagging of spermatozoa [22] and indicated a 10.6 % higher binding of sperm stored at 5 °C vs. 17 °C in a cold-shock protecting extender [2].

The aim of the present study was to examine whether chilling boar semen to 5 °C and subsequent storage for 24 h at this temperature in two different extender media affects fertility-relevant sperm traits, i.e., the subpopulation structure in motile spermatozoa with respect to movement patterns and the ability of sperm to bind to oviduct explants *in vitro*. Overall, the value of the methods to sensitively estimate chilling injury after different semen treatments was evaluated.

## 2. Materials and Methods

### 2.1. Chemicals

All chemicals used were of analytical grade. They were purchased from Merck (Darmstadt, Germany) and Roth (Karlsruhe, Germany), unless otherwise stated. Propidium iodide (PI) was obtained from Sigma-Aldrich (Steinheim, Germany), fluorescein-isothiocyanate conjugated peanut agglutinin (PNA-FITC) from Axxora (Lörrach, Germany), whereas MitoTracker^®^ Green FM and MitoTracker^®^ Red FM were obtained from Life Technologies (Darmstadt, Germany).

### 2.2. Semen Collection and Processing

Ejaculates were used from seven mature, healthy boars (Pietrain, Duroc and crossbred animals) housed at the Unit for Reproductive Medicine. Boars were kept and handled in accordance with the European Commission Directive for pig welfare. Semen collection was performed routinely by trained personnel using the “gloved-hand” method. Only normospermic ejaculates were used, corresponding to ≥70% motile, ≤25% morphological abnormal sperm, and ≤15% spermatozoa with cytoplasmic droplets. Each ejaculate was split into two equal volumes and diluted in either pre-warmed (32 °C) Beltsville Thawing Solution (BTS) or Androstar^®^ Plus extender (both from Minitüb, Tiefenbach, Germany) to a concentration of 20 × 10^6^ spermatozoa/mL. Two subsamples from each portion were stained with MitoTracker^®^ Green FM (MTgreen), and another two subsamples with MitoTracker^®^ Red FM (MTred). Control samples were processed with DMSO. Staining of spermatozoa followed a previously established protocol [22]. One hundred milliliters of diluted sperm from a given extender was mixed with either MTgreen stock solution (200 nM final concentration), MTred stock solution (200 nM final concentration) or 10 µL DMSO (solvent control) and incubated for 15 min at 38 °C. To remove excess dye, 50 mL diluted semen was layered on top of 5 mL 20% Percoll^®^ working solution in a centrifugation tube with a conical bottom and centrifuged for 10 min at 300 g max followed by 10 min at 750 g max. The 20% isosmotic Percoll^®^ working solution (pH 7.40 ± 0.05 at room temperature; 300 ± 5 mOsmol/kg;) was prepared by diluting pure Percoll^®^ (GE Healthcare, Uppsala, Sweden) with a HEPES-buffered saline medium (HBS; 137 mM NaCl, 20 mM HEPES, 10 mM Glucose, 2.5 mM KOH, pH 7.60 ± 0.05 at room temperature, 300 ± 5 mOsmol/kg) according to Vincent and Nadeau [23]. After centrifugation, the supernatant was aspirated, and the residual sperm pellet diluted in 20 mL isothermic extender (BTS or Androstar^®^ Plus) including 10% homologous seminal plasma. Sperm concentration was adjusted to 20 × 10^6^ sperm/mL. After processing, sperm were checked for staining and motility. Semen samples of 40 mL were kept for 90 min at room temperature before being transferred to a 17 °C storage unit. Samples which were designated to be stored at 5 °C were kept for 60 min at 17 °C; thereafter, for 60 min at 10°C before being finally stored at 5 °C.

### 2.3. CASA Assessment of Sperm Kinematics

An aliquot of 4 mL diluted semen was incubated for 15 min at 38 °C in a water bath and motility assessed with the CASA-system SpermVision^®^ (Minitüb). Four-chamber slides (Leja, Nieuw Vennep, The Netherlands) with a chamber depth of 20 µm were used. The CASA-system was equipped with a 20-fold objective lens, a camera adaptor (U-PMTVC tv-0.75; Olympus, Hamburg, Germany) and a camera with a resolution of 800 × 600 pixels (AccuPixel TM6760 CL; JAI, Glostrup, Denmark). It was operated by SpermVision software (Version 3.5; Minitüb) at 60 frames per second and 0.5-s recording time for each field of view. The percentage of motile (total motility) and progressively motile sperm (progressive motility) were recorded from 10 successive fields per sample. For progressively motile sperm the average straight-line velocity (VSL), curved-line velocity (VCL), average path velocity (VAP), linearity (LIN = VSL/VCL), straightness (STR = VSL/VAP), wobble (WOB = VAP/VCL), average amplitude of lateral head-displacement (ALH), and beat cross frequency (BCF) were assessed. A spermatozoon was considered to be motile when its average head orientation change (AOC) was higher than 2.5° and considered to be progressively motile when the distance moved from A to B in a straight line (DSL) exceeded 4.5 µm in 0.5 s [15].

### 2.4. Assessment of Plasma and Acrosomal Membrane Integrity

The percentage of sperm with intact plasma membrane (PI negative, PI neg.) and intact acrosomal membrane (PNA-FITC-negative, PNA-FITC neg.) was determined as described in Henning et al. [22]. Briefly, a 5-mL subsample of diluted semen was transferred to 995 mL HEPES-buffered saline medium containing propidium iodide (PI; final concentration 5 mg/mL), FITC-conjugated peanut agglutinin (PNA-FITC; final concentration 3.0 mg/mL), and Hoechst 33,342 (final concentration 0.75 mg/mL). After incubation (15 min, 25 °C), 10,000 events were analyzed using FloMax v2.4 software (Partec).

### 2.5. Sperm Binding in the cOEA

After 24 h storage, the spermatozoa’s ability to bind to oviduct epithelial cells *in vitro* was evaluated in a competitive oviduct explant assay. Oviductal explants were prepared from slaughter tissue of sows as described by Henning et al. [22]. For each ejaculate, a total of 16 explants, eight from each of two sows, were prepared. Single explants from two different sows were placed in separate wells of a 24-well plate. Each well contained 490 µL of pre-warmed and equilibrated Tyrode’s medium (38 °C; 5% CO_2_, 100% humidity). An equal number of spermatozoa (1 × 10^5^ sperm, i.e., 5 µL) preserved in BTS or Androstar^®^ Plus at the same storage temperature (17 °C or 5 °C) was added to one explant. Two explants per sow were used to compare spermatozoa extended in BTS (MTgreen; 17 °C) with sperm extended in Androstar^®^ Plus spermatozoa (MTred; 17 °C) as well as vice versa (BTS, MTred, 17 °C versus Androstar^®^ Plus, MTgreen, 17 °C). Details of the cross-over design are depicted in Figure S1. Likewise, sperm-oviduct binding for semen samples stored at 5 °C was evaluated.

Spermatozoa and explants were co-incubated in a CO_2_-incubator (38 °C, 5% CO_2_, 100% humidity). After 45 min, explants were gently washed in Tyrode’s medium to remove loosely bound spermatozoa. Explants were mounted on microscope slides in 60 µL of Tyrode´s medium surrounded by a frame of silicon grease (silicon stopcock grease, Dow Corning, Wiesbaden, Germany). The drop was sealed with a cover slip (20 × 20 mm).

Explants were then examined under an Olympus BX 41 microscope equipped with a heated stage, mercury lamp (100 Watt) and a filter set for simultaneous excitation and assessment of signals from MitoTracker^®^ Red and MitoTracker^®^ Green (F66-412, AHF Analysentechnik, Tübingen, Germany). A video of the explant was recorded at 200× magnification (long distance lens LUCPlanFL N, 20x/0.45 Ph1 ∞/0-2/FN 22) using a DP72 camera (Olympus, Hamburg, Germany) and CellP software (version 3.4; Olympus). Sperm binding was recorded as described previously (Henning et al. 2019). Video files were analyzed using CellP software. The explant area was measured and the number of fluorescent spermatozoa from each extender counted.

The average number of bound spermatozoa per 1 mm^2^ was defined as binding index in analogy to Petrunkina et al. [24]. The binding index (BI) was calculated as follows:

BI(E1) = (Nl1 + Nl2 + Nl3)/(Al1 + Al2 + Al3)

BI(E2) = (Nl1 + Nl2 + Nl3)/(Al1 + Al2 + Al3)

where

E1, E2 = explant from sow no. 1, explant from sow no. 2
Al1, Al2, Al3 = Area of location no. 1, 2, and 3, respectively
Nl1, Nl2, Nl3 = number of spermatozoa at location no. 1, 2, and 3, respectively

The binding index for each combination of extender × storage temperature × MitoTracker^®^ color was calculated as follows:

BI (BTS, 17 °C, MTgreen) = (BI(E1) + BI (E2))/2

The use of differentially tagged sperm (MitoTracker^®^ Red or MitoTracker^®^ Green) on the same oviduct explant allowed a direct comparison of how many spermatozoa from each extender bound per explant area [22]. The percentage of bound sperm per explant for each of the two extenders was calculated by dividing the number of spermatozoa for a given extender by the total number of bound spermatozoa:

%spermBTS (E1) = (NBTS)/(NBTS + NAndrostar^®^ Plus) * 100

where

%spermBTS = percentage of bound sperm from the extender BTS
E1 = explant from sow no. 1
NBTS + NAndrostar^®^ Plus = number of spermatozoa from BTS or Androstar^®^ Plus

In a second step the percentage of bound sperm from two explants was averaged:

%spermBTS (E1) = (%spermBTS (E1) + %spermBTS (E2))/2

where

E1, E2 = explant from sow no. 1, explant from sow no. 2

Additionally, the mean ratio of bound sperm was calculated in the competitive approach for samples stored at 5 °C or 17 °C.

### 2.6. Statistical Analysis

Data were analyzed using Excel^®^ and the Statistical Analysis System software (SAS^®^, version 9.2; SAS Inst. Inc., Cary, NC, USA). Data were tested for normal distribution (Shapiro-Wilk test; PROC UNIVARIATE). Where appropriate, data from individual parameters were logarithmically or square root transformed to achieve normality. Influence of storage temperature and extender were tested by ANOVA for repeated measurements (PROC GLM). The factor MitoTracker^®^ color was included in the ANOVA calculations for CASA parameters. Comparisons between extender by temperature combinations were made with Student’s *t*-test for paired observations (PROC UNIVARIATE). Data for which no normal distribution could be achieved, i.e., total motility and progressive motility, were analyzed with the Friedman test for influence of MitoTracker^®^ staining, storage temperature, and extender (PROC FREQ), followed by pair-wise comparisons with Wilcoxon signed-rank test (PROC UNIVARIATE). Cluster analysis based on CASA parameters from all motile spermatozoa (*n* = 24,032 sperm) was performed by hierarchical analysis using squared Euclidian distance as the distance measurement and the ‘centroid’ algorithm for cluster fusion (PROC CLUSTER) as described by Henning et al. [15]. The choice of a suitable solution from the clustering procedure was guided by the cubic clustering criterion (CCC), pseudo-F statistics and pseudo t^2^ values. The aim was to select a solution which explains a high degree of variation in the data set without fragmenting the data into too many subunits whose biological meaning cannot be interpreted anymore. An effect of storage temperature was estimated with a ‘χ^2^’-test for homogeneity separately for each extender and the effect size was estimated based on Cramer’s V (PROC FREQ). Cramer’s V (range 0 to 1) was interpreted according to the guidelines from Cohen [25]: V < 0.10 = no effect, 0.10 < V ≤ 0.30 = small effect, 0.30 < V ≤ 0.50 = moderate effect. Correlations between the binding index, i.e., number of bound spermatozoa per 1 mm^2^, and semen parameters were estimated with Spearman´s rank correlation coefficient (PROC CORR) based on data that were derived from all boars and all extender by storage temperature combinations. Non-linear regressions between the binding index and selected parameters were calculated (PROC NLIN). The effect of the storage temperature on the sperm binding index for samples stored at 5 °C or 17 °C from individual boars was evaluated by a Kruskal-Wallis test. Unless otherwise stated, data are presented as mean ± standard deviation. Differences were considered to be significant when their occurrence probability was less than 5% (*p* < 0.05).

## 3. Results

### 3.1. Sperm Kinematics

Staining with MitoTracker^®^ dyes had no influence on any sperm motility parameter (*p* > 0.05; Table S1). Therefore, CASA parameters from three measurements (MitoTracker^®^ Green-stained sperm, MitoTracker^®^ Red-stained sperm, and DMSO treated sample) were averaged for each extender by storage temperature combination. Sperm storage temperature and extender had a significant influence on total and progressive sperm motility (both *p* < 0.05).

**Figure 1:**
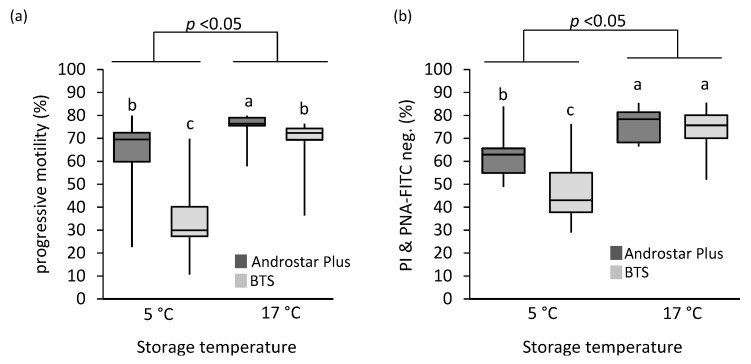
Effect of storage temperature and semen extender on progressive motility and viability of boar spermatozoa. Storage temperature (5 °C or 17 °C) and semen extender (Beltsville Thawing Solution (BTS) or Androstar^®^ Plus) had a significant impact on (**a**) progressive motility and (**b**) the number of viable spermatozoa with intact acrosomal membrane (PI & PNA-FITC negative) after 24 h preservation. Different lower-case superscripts indicate significant differences based on Wilcoxon signed-rank test (**a**) or Student’s *t*-test for paired samples (**b**; all *n* = 7 boars; all *p* < 0.05).

Total motility and sperm velocity at 17 °C and 5 °C were higher in sperm stored in Androstar^®^ Plus when compared to BTS (Figure 1a and Table 1). Chilling spermatozoa in BTS to 5 °C significantly reduced the average curvi-linear velocity and beat cross frequency in the progressive motile sperm population (*p* < 0.05), which was either not or at a less significant extent, observed for spermatozoa stored in Androstar^®^ Plus (Table 1).

### 3.2. Plasma Membrane and Acrosome Integrity

Both, storage temperature and extender, had a significant influence on sperm membrane integrity (*p* < 0.05). While no difference between extenders was detectable for sperm stored at 17 °C, the percentage of spermatozoa with intact plasma and acrosome membrane after 5 °C storage was higher in samples diluted in Androstar^®^ Plus (Figure 1b).

### 3.3. Cluster Analysis

**Figure 2:**
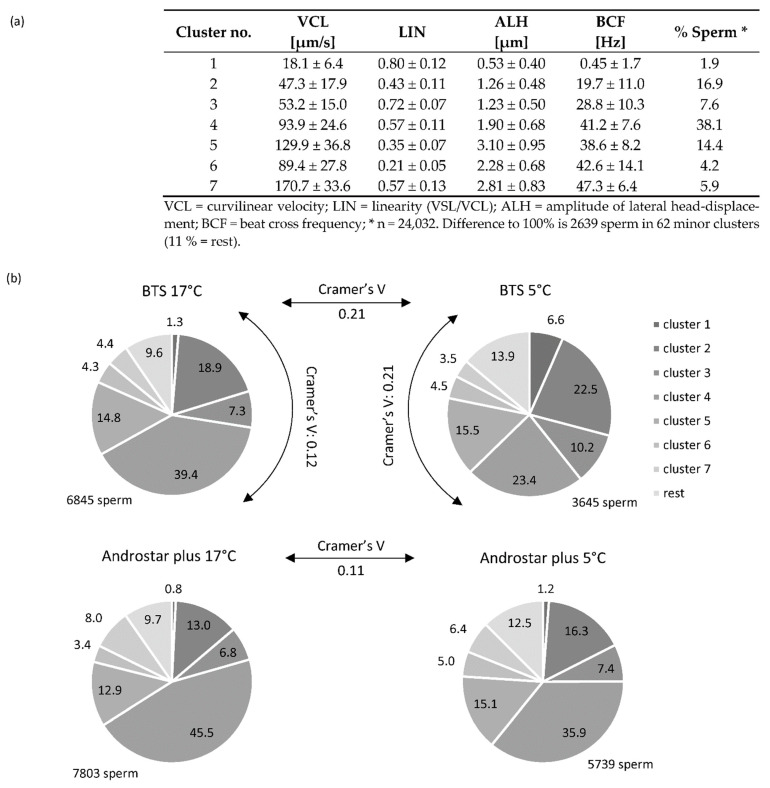
Effect of storage temperature and semen extender on the distribution of sperm in seven major kinematic clusters. (**a**) Subpopulations of motile spermatozoa as defined by cluster analysis of single sperm data from all motile cells. (**b**) The subpopulations structure for samples stored for 24 h at 17 °C or 5 °C in either Beltsville Thawing Solution (BTS) or Androstar^®^ Plus. All values are percentages. Chilling to 5 °C resulted in a significant change in sperm distribution to the different subpopulations, i.e., cluster (*p* < 0.05). Cramer’s V indicates the effect size of the different storage temperatures or the semen extender on the distribution of the sperm to the different subpopulations (range: 0–1). The following guidelines were used for interpretation: V < 0.10 = no effect, 0.10 < V ≤ 0.30 = small effect, 0.30 < V ≤ 0.50 = moderate effect.

From the cluster analysis procedure, a solution explaining 68% of the variance in the data set was chosen, which consisted of seven major sperm subpopulations, i.e., clusters (Figure 2a). These major clusters were comprised of 89% of the single spermatozoa. The dominant motility pattern was spermatozoa with relatively average velocity, LIN, ALH, and BCF (Cluster 4; 38.1% of all spermatozoa), followed by spermatozoa with half of the velocity of Cluster 4 and significantly lower LIN, ALH, and BCF (Cluster 2; 16.9%), and by fast spermatozoa with low linearity, but high ALH (Cluster 5; 14.4%). A stratified view of the data is presented in Figure 2b. Storage temperature had a significant effect on the subpopulation structure in the motile sperm population (*p* < 0.05; Figure 2b). The effect size of storage at 5 °C, as estimated by Cramer’s V, was higher in the BTS extender (0.21) than in the Androstar^®^ Plus extender (0.11). The cell chi-squared values indicated that the chilling-induced changes in sperm motility patterns in the BTS extender were predominantly caused by a reduction in the sperm population with average speed, linearity, and BCF (Cluster 4; 17 °C: 39.4 %, 5 °C: 23.4%) and a concomitant increase in sperm subpopulations with low velocity and low ALH and BCF values (Clusters 1, 2, and 3).

The subpopulation structure of motile spermatozoa differed between extenders (*p* < 0.05; Figure 2b). The effect size was higher at 5 °C (BTS vs. Androstar^®^ Plus: 0.21) than at 17 °C (0.12). The difference at 5 °C was mainly caused by fewer sperm with very low velocity, ALH, and BCF (Cluster 1) and more sperm with average speed, linearity, and BCF (Cluster 4) in Androstar^®^ Plus than in BTS.

### 3.4. Sperm Binding to Oviduct Explants

**Figure 3:**
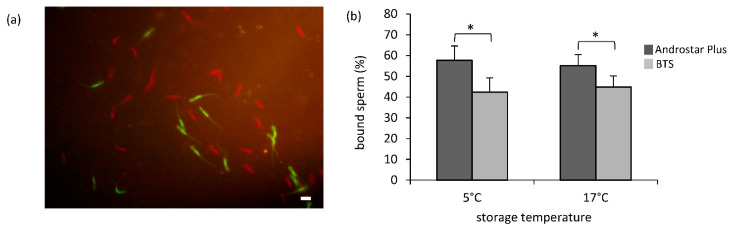
Effect of storage temperature and semen extender on sperm binding in the competitive oviduct explant assay. A competitive oviduct explant assay was applied to directly compare the ratio of oviduct-bound spermatozoa for semen samples which had been stored for 24 h in either Beltsville Thawing Solution (BTS) or Androstar^®^ Plus at 5 °C or 17 °C. (**a**) Exemplary image from video recording of MitoTracker^®^ Green- or MitoTracker^®^ Red-tagged sperm bound to the oviduct explant surface (scale bar = 20 µm). (**b**) The semen extender had a significant impact on the percentage of sperm that were bound to oviduct explants. An asterisk (*) indicates significant differences between the percentage of bound sperm per extender at a given storage temperature (*n* = 7 boars; *p* < 0.05).

Staining of spermatozoa with different MitoTracker^®^ dyes allowed a direct comparison of the relative amount of sperm per explant area (Figure 3a). Independent of storage temperature, the number of bound spermatozoa was influenced by the semen extender (*p* < 0.05). Relatively more spermatozoa were bound per explant area when samples had been preserved in Androstar^®^ Plus at 5 °C (58 ± 6%) instead of BTS (42 ± 6%; *p* < 0.05; Figure 3b). The difference was less prominent albeit significant for samples stored at 17 °C (55 ± 6% vs. 45 ± 6%; *p* < 0.05; Figure 3b). The number of spermatozoa bound per explant area tended to be influenced by storage temperature (*p* = 0.052) and varied considerably between samples from individual boars (Figure S2).

### 3.5. Correlation of Sperm Parameters and Number of Bound Sperm

**Figure 4:**
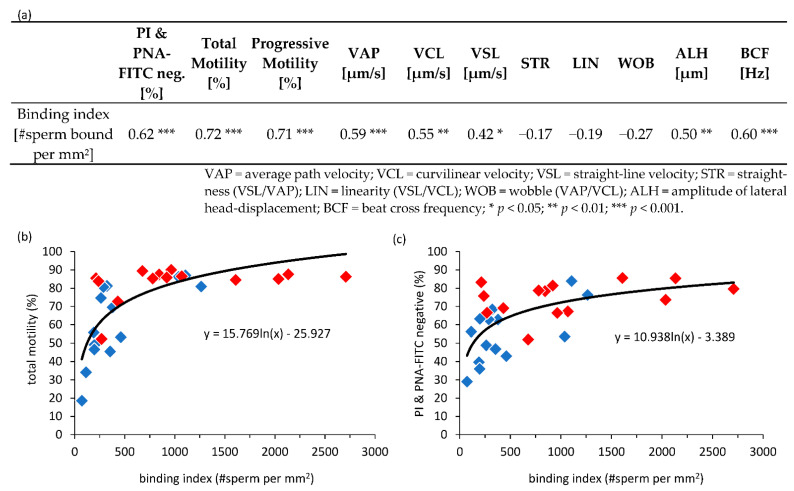
Correlation between semen parameters and sperm oviduct binding of sperm stored for 24 h in either Beltsville Thawing Solution or Androstar^®^ Plus at 5 °C or 17 °C. (**a**) Spearman correlation coefficients between the number of spermatozoa bound per surface area (binding index), CASA parameters and the number of viable spermatozoa with intact acrosomes (PI & PNA-FITC negative; *n* = 28 samples). The relation between the binding index and total motility (**b**) as well as the percentage viable spermatozoa with intact acrosomes (**c**) was best modeled by a logarithmic function. Red diamonds represent data from samples stored at 17 °C. Blue diamonds represent data from samples stored at 5 °C.

The number of bound spermatozoa per 1 mm^2^ correlated positively with the percentage of viable, acrosome-intact spermatozoa (r = 0.62, *p* < 0.001) and the percentage of motile spermatozoa (r = 0.72, *p* < 0.001; *n* = 28; Figure 4a). The relation between these parameters was modelled best by logarithmic regression curves (Figure 4b,c).

Average sperm velocity, amplitude of lateral head-displacement, and beat cross frequency for progressive motile spermatozoa all positively correlated with the sperm binding index (Figure 4a). When using results from cluster analysis instead of average CASA parameters, only the amount of sperm in Cluster 1 correlated negatively with the binding index (r = −0.43, *p* < 0.05). Cluster 1 was composed of sperm with very low speed and vigor in sperm movement.

## 4. Discussion

The present study demonstrates that analysis of movement patterns in motile boar spermatozoa and competitive sperm-oviduct binding *in vitro* provide sensitive information on chilling-induced sublethal changes in sperm function. Noteworthy, the assays were sufficiently sensitive to reveal the impact of different extender media on the extent of chilling injury. Thus, the assays used here provide additional information to established descriptors of chilling effects in viable spermatozoa, such as mitochondria membrane potential, apoptotic-like changes, membrane fluidity, calcium influx, and changes in tyrosine phosphorylation (c.f. [26,27,28,29]).

Analysis of mean values from CASA parameters was appropriate for determining chilling effects on motility and progressive motility but underestimated the effects on flagellar tracks and beating intensities in the motile sperm population by revealing only few significant effects. Seen as advantageous, cluster analysis considered multiple kinematic traits for grouping motile spermatozoa simultaneously, many of them (e.g., VCL, LIN, ALH, BCF) with an established association to fertility (reviewed in [30]). The results from the cluster analysis demonstrated that chilling caused a redistribution of motile spermatozoa to the identified clusters, with a decrease in the subpopulations displaying a faster and more vigorous movement. Clustered data were sufficiently sensitive to display the influence of the semen extender on the chilling stress, although higher frame rates of 200 frames per second could be favorable to further improve the sensitivity of this approach [31]. The degree of cell death and the subtler influence of chilling on the subpopulation structure in motile sperm were both attenuated by the use of the long-term extender Androstar^®^ Plus which augments previous results on the sperm-protective attributes of this medium [28,29,32]. Noteworthy, more than 45 % of the motile spermatozoa from samples stored in the short-term extender BTS at 5 °C showed relatively high speed and beat cross frequency (Cluster 4 to 7), indicating that energy provision and the flagellar machinery are not greatly affected by chilling.

The present study also demonstrates that the cOEA sensitively detects extender effects of sperm chilled to 5 °C and even after storing at 17 °C for 24 h. Storing spermatozoa at 5 °C instead of 17 °C increased the relative difference in bound sperm between the two extenders, which indicates a higher capacity of Androstar^®^ Plus to preserve the spermatozoa’s ability for formation of the female sperm reservoir. The binding index correlated positively with average sperm motility and the percentage of viable, acrosome-intact spermatozoa. This suggests that viable spermatozoa which actively swim in a directed fashion have a higher likelihood of reaching the explant surface and encountering the oviductal glycan binding sites, even though the spermatozoa are deposited in close vicinity to the oviductal explants. This assumption is further supported by the negative correlation of the binding index and the magnitude of a sperm cluster representing cells with very low speed and vigor in sperm movement. The discrepancy to earlier reports that did not observe a correlation between the binding index and sperm motility or viability may be explained by an increased sensitivity of the competitive assay approach [19,24,33]. The competitive approach reduces the bias of the female tissue and enables a reliable detection of fluorescent-labeled, bound spermatozoa. Moreover, the wide spread in basic sperm quality traits between the samples used here made it more likely to detect such correlations.

Notably, the correlation between sperm quality traits and the binding index was not perfectly linear. Storage at 5 °C reduced the number of bound spermatozoa to a larger extent (2.3-fold) than what could have been expected from the decline in sperm motility and membrane integrity for samples stored in either extender (1.2 to 1.6-fold). This indicates that other sperm characteristics were affected by chilling stress. In fact, sperm binding to oviductal epithelial cells is regarded as a highly selective process that requires specific functional properties of the spermatozoa [34,35,36], thus explaining the predictive value of quantifying sperm-oviduct binding for fertility in pigs and cows [37].

A further observation was that although chilling to 5 °C considerably diminished the number of spermatozoa bound to the oviduct explants, the decline appeared not to be evident for all boars. This may be due to inter-male differences in membrane lipid composition, which have been associated with cryosurvival or other intrinsic sperm properties [38]. To determine whether the observed difference in the current study was boar-specific, was, however, beyond the scope of this study.

Noteworthy, in the present study, chilling stress was higher compared to a recently established hypothermic storage concept using a cold-shock protective extender (Androstar^®^ Premium) and an adapted cooling regime [2,39]. Regardless of the degree of chilling stress, our recent multi-color flow cytometry studies targeting mitochondria membrane potential, membrane fluidity, and the response to a capacitation stimulus in viable sperm revealed that surviving spermatozoa have a high resistance to subsequent storage stress and maintain a high fertilizing capacity *in vivo* [40,41]. Together with the results of the present study, we suggest that for boar semen storage at 5 °C to be realized in practice, a sufficiently high number of (viable) sperm that colonize the oviductal sperm reservoir is crucial.

For the appropriate adaptation of sperm numbers in the insemination dose, the assessment of motility traits in combination with kinematic movement patterns will be a useful tool. It has to be considered that the ability of sperm to traverse the uterus and to cross the physical barriers of the uterotubal junction, to bind, and to release/to be released from the oviductal epithelial cells might require different spatiotemporal kinematic characteristics. For this reason, threshold values for kinematic means or preformation of cluster characteristics are inadvisable. New methods in semen processing should always be compared with established methods for which key data for the fertilizing ability of the sperm are already available. Not only the total number of motile spermatozoa should be compared *in vitro*, but also the distribution of motile sperm to individual subpopulations.

## 5. Conclusions

In conclusion, chilling of extended boar semen to 5 °C and subsequent storage for 24 h at this temperature alters the subpopulation structure of the motile cells and concomitantly reduces the number of spermatozoa capable of binding to the oviduct epithelium *in vitro*. A competitive oviduct explant assay sensitively highlights that the choice of semen extender influences the degree of chilling injury and shows to what extent the sperm-oviduct binding ability is preserved. The identification of subpopulation shifts in sperm kinematics and competitive sperm oviduct binding *in vitro* present meaningful biological tests to assess novel semen preservation strategies. These are helpful tools on the way towards hypothermic semen storage for reducing the antibiotic use in pig reproduction.

## Figures and Tables

**Table 1:** Comparison of CASA kinematic parameters for semen samples after 24 h storage in Beltsville Thawing Solution (BTS) or Androstar^®^ Plus at 17 °C or 5 °C (*n* = 7 boars). Different superscripts (a–d) within a row indicate significant differences. Results are based on Student’s *t*-test for paired samples unless otherwise indicated (*p* < 0.05). Data are presented as mean and standard deviation.

	BTS		Androstar^®^ Plus	
	5 °C	17 °C	5 °C	17 °C
total motility [%] *	49.9 ± 18.4 ^a^	80.9 ± 12.7 ^b^	73.4 ± 18.4 ^c^	85.2 ± 5.6 ^d^
progressive motility [%] *	35.1 ± 18.5 ^a^	67.5 ± 14.0 ^b^	62.4 ± 19.0 ^b^	74.7 ± 7.7 ^c^
VAP [µm/s]	44.6 ± 12.6 ^a^	54.6 ± 7.1 ^b^	58.8 ± 7.7 ^b,c^	62.2 ± 5.6 ^c^
VCL [µm/s]	73.7 ± 28.4 ^a^	91.7 ± 15.6 ^b^	99.1 ± 20.1 ^b,c^	100.9 ± 15.9 ^c^
VSL [µm/s]	33.1 ± 7.6 ^a^	41.6 ± 5.6 ^b^	43.0 ± 5.6 ^b^	48.3 ± 3.8 ^c^
STR	0.75 ± 0.09 ^a, b^	0.76 ± 0.06 ^a^	0.73 ± 0.07 ^b^	0.77 ± 0.05 ^a,b^
LIN	0.49 ± 0.12	0.46 ± 0.07	0.44 ± 0.10	0.48 ± 0.08
WOB	0.62 ± 0.09 ^a^	0.60 ± 0.05 ^a,b^	0.60 ± 0.08 ^b^	0.62 ± 0.06 ^a,b^
ALH [µm]	1.78 ± 0.69 ^a^	2.11 ± 0.47 ^a,b^	2.22 ± 0.53 ^b^	2.21 ± 0.45 ^b^
BCF [Hz]	25.8 ± 6.3 ^a^	33.6 ± 3.0 ^b^	33.9 ± 3.8 ^b^	37.1 ± 2.2 ^c^

VAP = average path velocity; VCL = curvilinear velocity; VSL = straight-line velocity; STR = straightness (VSL/VAP); LIN = linearity (VSL/VCL); WOB = wobble (VAP/VCL); ALH = amplitude of lateral head-displacement; BCF = beat cross frequency; * Wilcoxon signed-rank test.

## Data Availability

The data presented in this study are available on request from the corresponding author.

## Supplementary Materials

The following supporting information can be downloaded at: https://www.mdpi.com/article/10.3390/ani12060712/s1, Figure S1: Experimental design; Figure S2: Effect of storage temperature on sperm binding for individual boars; Table S1: Effect of staining with MitoTracker^®^ dyes on CASA kinematic parameters of boar spermatozoa.
