# Supplementary material for: Assessment of Chilling Injury in Boar Spermatozoa by Kinematic Patterns and Competitive Sperm-Oviduct Binding In Vitro
Source: Animals (Basel). 2022 Mar 11;12(6):712. doi: 10.3390/ani12060712 (PMC8944792; doi:10.3390/ani12060712)
Supplement: Supplementary file 1 [file animals-12-00712-s001.zip › animals-1607194-supplementary.pdf]

Table S1.

Effect of staining with MitoTracker® dyes on CASA kinematic parameters of boar spermatozoa.

Comparison of CASA parameters for semen samples extended in Beltsville Thawing Solution (a) or Androstar® Plus (b). Samples were treated with either MitoTracker® Green FM, MitoTracker® Red FM or DMSO, respectively, and stored at 17°C or 5°C. The staining of the samples had no influence on the CASA parameters ($p > 0.05$). Data are shown as mean and standard deviation.

**(a)**		BTS 5 °C			BTS 17 °C	
	MitoTracker® Red	MitoTracker® Green	DMSO	MitoTracker® Red	MitoTracker® Green	DMSO
Total motility [%]	48.1 ± 18.3	48.6 ± 19.9	53.2 ± 18.1	79.4 ± 16.2	80.5 ± 11.9	82.8 ± 10.9
Progressive motility [%]	34.4 ± 20.0	32.5 ± 19.0	38.3 ± 18.2	65.3 ± 21.7	66.4 ± 12.6	70.8 ± 10.0
VAP [µm/s]	44.3 ± 15.6	49.1 ± 15.8	49.1 ± 11.7	51.6 ± 11.0	53.4 ± 8.2	58.8 ± 6.9
VCL [µm/s]	73.2 ± 32.9	66.3 ± 34.3	81.7 ± 26.3	83.5 ± 21.6	90.3 ± 19.0	101.3 ± 20.9
VSL [µm/s]	32.8 ± 9.3	30.2 ± 10.2	36.4 ± 8.0	40.3 ± 7.8	40.7 ± 6.1	43.6 ± 5.2
STR	0.75 ± 0.09	0.76 ± 0.11	0.74 ± 0.07	0.78 ± 0.05	0.76 ± 0.07	0.74 ± 0.09
LIN	0.50 ± 0.15	0.46 ± 0.10	0.49 ± 0.09	0.49 ± 0.08	0.46 ± 0.08	0.44 ± 0.11
WOB	0.62 ± 0.10	0.64 ± 0.11	0.61 ± 0.08	0.59 ± 0.07	0.59 ± 0.06	0.62 ± 0.07
ALH [µm]	1.79 ± 0.75	1.64 ± 0.79	1.90 ± 0.67	1.93 ± 0.48	2.13 ± 0.59	2.29 ± 0.58
BCF [Hz]	26.1 ± 8.5	23.1 ± 8.4	28.3 ± 6.4	32.0 ± 5.7	33.2 ± 3.1	35.6 ± 2.1

VAP = average path velocity; VCL = curvilinear velocity; VSL = straight-line velocity
STR = straightness (VSL/VAP); LIN = linearity (VSL/VCL); WOB = wobble (VAP/VCL)
ALH = amplitude of lateral head-displacement; BCF = beat cross frequency

(b)

	Androstar® Plus 5 °C			Androstar® Plus 17 °C		
	MitoTracker® Red	MitoTracker® Green	DMSO	MitoTracker® Red	MitoTracker® Green	DMSO
Total motility [%]	72.9 ± 18.5	73.8 ± 17.3	73.4 ± 19.8	84.2 ± 8.2	85.4 ± 7.1	85.9 ± 2.9
Progressive motility [%]	61.4 ± 20.5	62.1 ± 18.6	63.9 ± 19.0	72.8 ± 12.8	73.3 ± 9.2	78.1 ± 3.3
VAP [µm/s]	58.9 ± 10.1	58.1 ± 8.7	59.4 ± 6.0	59.0 ± 10.5	62.1 ± 5.0	65.6 ± 7.7
VCL [µm/s]	99.6 ± 23.0	99.0 ± 21.4	98.5 ± 18.5	95.6 ± 23.1	101.9 ± 11.6	105.4 ± 18.4
VSL [µm/s]	43.0 ± 6.8	42.8 ± 5.6	43.3 ± 5.0	45.6 ± 7.2	48.2 ± 4.5	51.1 ± 5.5
STR	0.73 ± 0.07	0.73 ± 0.07	0.73 ± 0.08	0.77 ± 0.06	0.77 ± 0.05	0.78 ± 0.05
LIN	0.44 ± 0.10	0.44 ± 0.10	0.45 ± 0.10	0.49 ± 0.09	0.47 ± 0.08	0.49 ± 0.08
WOB	0.60 ± 0.08	0.59 ± 0.08	0.61 ± 0.07	0.62 ± 0.06	0.61 ± 0.06	0.63 ± 0.06
ALH [µm]	2.25 ± 0.63	2.25 ± 0.53	2.17 ± 0.47	2.21 ± 0.53	2.24 ± 0.40	2.17 ± 0.46
BCF [Hz]	33.8 ± 5.0	34.0 ± 3.7	34.0 ± 3.1	35.5 ± 4.7	37.6 ± 2.4	38.2 ± 3.0

VAP = average path velocity VCL = curvilinear velocity VSL = straight-line velocity
STR = straightness (VSL/VAP) LIN = linearity (VSL/VCL) WOB = wobble (VAP/VCL)
ALH = amplitude of lateral head-displacement BCF = beat cross frequency

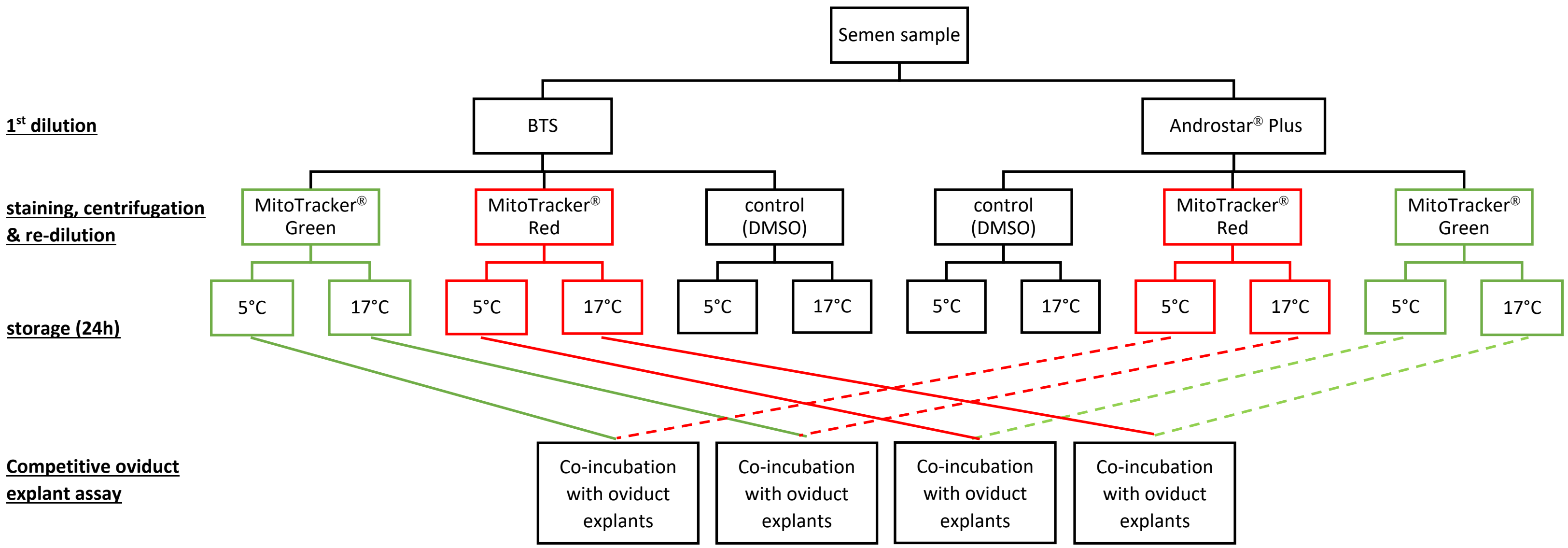

Figure S1.

Flow chart illustrating the sample processing before co-incubation with oviduct explants.

Boar semen samples were extended in Beltsville Thawing Solution (BTS) or Androstar® Plus extender. Details on the individual processing steps are provide in the Material and Methods section.

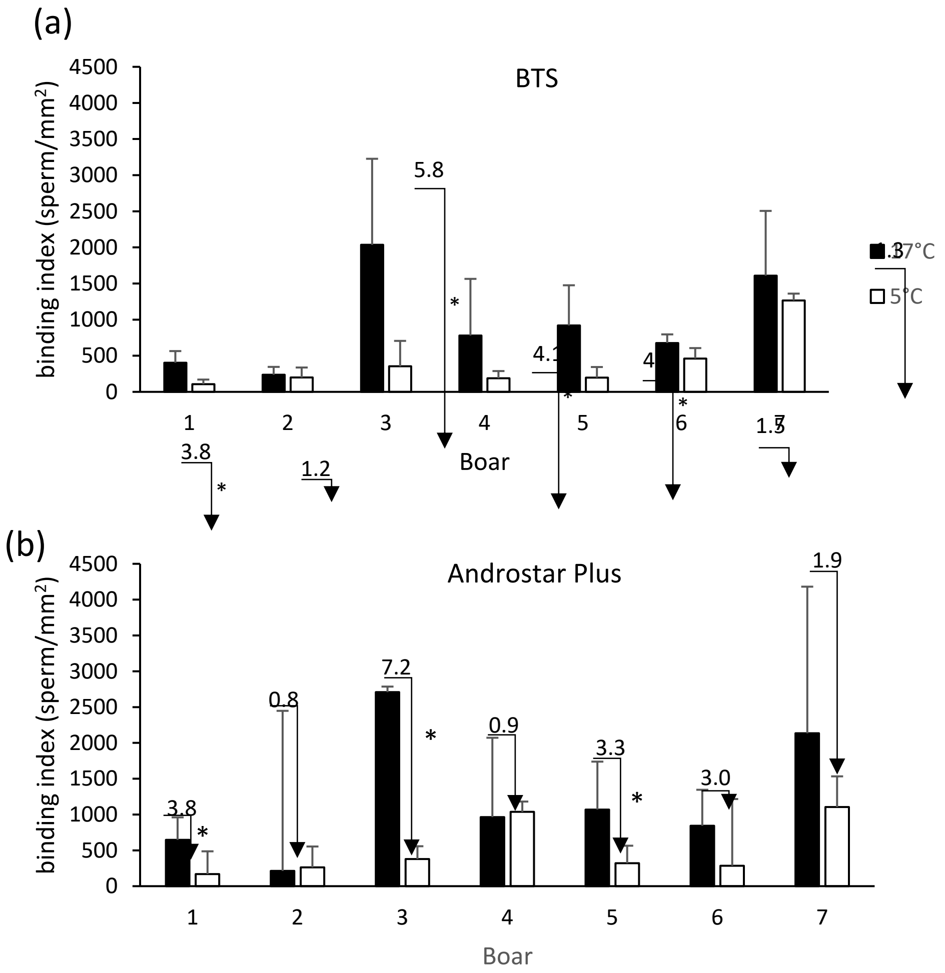

Figure S2. *Effect of storage temperature on sperm binding for individual boars.*
Number of bound spermatozoa per explant area (binding index) for semen samples from individual boars after storage in (a) Beltsville Thawing Solution (BTS) or (b) Androstar Plus at either 5°C or 17°C (n = 4 explants per condition). Numbers indicate the fold change in binding index. An asterisk indicates a significant effect of storage temperature on the binding index as estimated by Kruskal-Wallis test ($p < 0.05$).
